# AI-Driven Optimization of Cu_2_O Modified Bitumen: A Multi-Scale Evaluation of Rheological, Aging, and Moisture Susceptibility Performance

**DOI:** 10.3390/ma18174201

**Published:** 2025-09-08

**Authors:** Sebnem Karahancer

**Affiliations:** Department of Civil Engineering, Faculty of Technology, Isparta University of Applied Sciences, 100. Yil Campus, 32260 Isparta, Turkey; sebnemsargin@isparta.edu.tr

**Keywords:** Cu_2_O modified bitumen, AI modeling, binder performance, rheology, moisture susceptibility, gradient boosting, optimization, grid search

## Abstract

This study explores the integration of copper oxide (Cu_2_O) into bitumen and leverages Artificial Intelligence (AI) to evaluate and optimize the binder’s performance across multiple scales. Comprehensive laboratory tests, including conventional binder properties, rheological analysis, aging simulations, low-temperature cracking, and moisture susceptibility, were conducted on base and Cu_2_O modified asphalt binders. The results were used to train predictive models using gradient boosting regressors for each performance category. Optimization identified ideal Cu_2_O ratios for different engineering goals, offering practical recommendations. Based on this integrated cost-performance analysis, a Cu_2_O concentration of 2.3% was recommended as the most efficient trade-off point. AI modeling using Gradient Boosting Regressor (GBR) achieved high predictive performance, with R^2^ values reaching 0.98 for BBR prediction and 0.78 for rheology, and mean absolute error (MAE) values as low as 4.21. This demonstrates the model’s robustness in capturing complex nonlinear binder behaviors.

## 1. Introduction

Bitumen modification is a widely accepted technique for improving the durability and sustainability of pavements. Copper oxide (Cu_2_O) has emerged as a promising additive due to its antioxidative, filler, and anti-stripping properties [1]. However, the effects of varying Cu_2_O concentrations on binder performance are not well quantified, especially in the context of advanced modeling techniques.

Recent advances in sustainable asphalt modification have highlighted the use of bio-oil in combination with organosolv lignin, which significantly alters both rheological and chemical properties of bitumen. Zhang et al. (2022) demonstrated that such bio-based modifiers can enhance the viscoelastic behavior and oxidative stability of binders, providing a reference framework for nanomaterial-based modifications such as Cu_2_O [2].

Similarly, Chen et al. (2023) reported that high-viscosity binders containing warm-mix additives exhibit improved workability and enhanced rutting resistance without compromising fatigue performance. This emphasizes the importance of evaluating rheological responses under different modification strategies, comparable to the approach adopted in the present Cu_2_O study [3].

Ren et al. (2024) further underlined that chemical and rheological indicators should be jointly assessed to determine the efficiency of modified or rejuvenated binders. This aligns with the multi-parameter evaluation framework employed in the current work, where Cu_2_O dosage optimization was guided by rutting, cracking, and moisture susceptibility indices [4].

The performance of asphalt binders is directly related to their durability against temperature fluctuations and environmental conditions. Low-temperature performance is a critical factor affecting the cracking resistance of asphalt binders. For example, in a study conducted by Xu et al. [5], the evaluation of low-temperature performance was addressed using a dynamic shear rheometer (DSR). This study examines the effects of aging processes at low temperatures and demonstrates how the mechanical properties of binders change over time. In this context, the potential of Cu_2_O to improve the low-temperature performance of asphalt binders can be evaluated through such rheological tests [6].

Moreover, the effect of Cu_2_O on the aging resistance of asphalt binders is also crucial. In a study by Zhang et al. [7], the effects of long-term laboratory aging on the rheological properties of polymer-modified asphalt binders were investigated. This research highlights how aging impacts the viscoelastic properties of asphalt binders and how these changes contribute to the overall performance of asphalt. The potential of Cu_2_O to enhance the aging resistance of asphalt binders can be supported through similar testing.

Cu_2_O was selected over CuO and other oxides (Fe_2_O_3_, Cr_2_O_3_, MnO_2_) due to its proven antioxidative and anti-stripping performance, stability under bitumen processing conditions, and favorable cost–performance ratio [8].

Moisture susceptibility is another key factor affecting the performance of asphalt binders. In a study conducted by Karahancer, S. [8], the effects of Cu_2_O on the rutting and moisture sensitivity of asphalt binders were examined. This study reveals Cu_2_O’s potential to reduce moisture susceptibility in asphalt binders. Accordingly, Cu_2_O’s potential to enhance moisture resistance in asphalt binders can be assessed through such tests.

AI-based modeling is a powerful tool for predicting the performance of asphalt binders. In a study by Notani et al. [9], the high-temperature performance and activation energy of carbon-black-modified asphalt binders were evaluated. This study demonstrates the effectiveness of AI-based predictive models in evaluating the performance of asphalt binders. A similar approach can be adopted to predict the performance of Cu_2_O-modified asphalt binders.

Prediction models developed using gradient boosting regression provide high accuracy across each performance category. For instance, in a study by Aflaki and Hajikarimi [10], the mechanical and rheological properties of various modified asphalt binders were investigated. This research shows how different modifications influence the performance of asphalt binders and the predictability of these effects. A similar modeling approach can be used to optimize the performance of Cu_2_O-modified asphalt binders.

With the advancement of technology and data analytics, machine learning algorithms have become essential tools for modeling complex and non-linear relationships in various domains. In this context, the Gradient Boosting Regressor (GBR) algorithm was selected as the primary predictive model to capture the intricate associations between input features and target variables in this study. The choice of GBR was based on an extensive comparative analysis with other widely used machine learning models, such as Random Forest and Multi-Layer Perceptron (MLP). The results of these preliminary evaluations consistently demonstrated the superior predictive accuracy, stability, and generalization performance of GBR on both training and unseen test data [11].

Gradient boosting techniques are particularly well-suited for high-dimensional and heterogeneous datasets, as they iteratively minimize prediction errors through the sequential training of weak learners—typically decision trees. Advanced tree-based ensemble methods, such as XGBoost and Gradient Boosting Machine (GBM), have shown remarkable performance in various predictive modeling tasks. For instance, in the study by Sahin [12], XGBoost was found to outperform both Random Forest and GBM in forecasting natural disasters, underlining the effectiveness of gradient boosting methods in modeling highly non-linear and context-dependent patterns.

Beyond predictive accuracy, the robustness and generalization ability of GBR further strengthen its applicability in real-world settings. In a study by Gupta et al. [13] focused on detecting motor insurance fraud, the use of GBM resulted in outstanding performance metrics, achieving an F1 score of 98% and an accuracy of 99%. These findings highlight GBR’s capability to handle imbalanced and noisy datasets without overfitting, a critical advantage in industrial and engineering applications. The F1 score is the harmonic mean of precision and recall, providing a balanced measure of model accuracy.

While neural network-based models such as MLP are often praised for their ability to approximate complex functions, GBR has demonstrated superior performance in terms of both interpretability and reliability in several comparative studies. For example, Kumar et al. [14] conducted a systematic evaluation of gradient-based learning algorithms and reported that MLP occasionally underperformed, especially in terms of consistency and overfitting, compared to boosting techniques. These results further validate the decision to prioritize GBR as the core model in this study, especially considering the structured nature of the experimental data.

Moreover, the overall performance of GBR is highly dependent on hyperparameter optimization, which plays a vital role in enhancing both the model’s accuracy and its generalization capacity. In this regard, Chu et al. [15] demonstrated that advanced optimization strategies, such as fast gradient projection methods, significantly improve the model’s efficiency and predictive power. Applying similar hyperparameter tuning strategies in this study allowed for the fine-tuning of GBR, ensuring high-performance outcomes across all target metrics.

In conclusion, the potential of Cu_2_O to enhance the performance of asphalt binders can be evaluated through multi-scale laboratory tests and AI-based modeling. This study identifies the optimal proportions of Cu_2_O for improving asphalt binder performance and provides practical recommendations aligned with engineering objectives.

This study integrates laboratory testing with machine learning (ML) models to evaluate Cu_2_O-modified bitumen. The aim is twofold: (1) understand how Cu_2_O affects performance across aging, cracking, and moisture contexts, and (2) use AI to predict and optimize performance based on Cu_2_O concentration.

## 2. Materials and Methods

### 2.1. Materials

The base asphalt binder used in this study was obtained from Turkish Petroleum Refineries Corporation (TUPRAS) (Izmir, Turkey). Its rheological characteristics were evaluated through penetration, softening point, ductility, rotational viscosity (RV), dynamic shear rheometer (DSR), and bending beam rheometer (BBR) tests. Based on these evaluations, the Performance Grade (PG) of the base binder was classified as PG 64-22. Detailed properties of the bitumen are provided in Karahancer, S. [8].

The blending process of the base binder with the Cu_2_O nanoparticles was carried out using a high-shear mixer operating at 160 °C, 3000 rpm for a duration of one hour to ensure proper dispersion.

### 2.2. Test Methods

#### 2.2.1. Rutting Resistance of Asphalt Binder

The complex modulus (G*) of asphalt binders, which reflects their resistance to deformation, was measured using a Dynamic Shear Rheometer (DSR). This test applies a sinusoidal shear stress to determine both elastic and viscous behavior. The G* value and the phase angle (δ) together describe the relative proportions of elastic and viscous components and are influenced by temperature and loading frequency. Tests were conducted using 25 mm parallel plates with a 1 mm gap. The samples were subjected to 0.1% strain at a frequency of 10 rad/s (1.59 Hz), starting from 52 °C with increments of 6 °C. After simulating short-term aging with the Rolling Thin Film Oven Test (RTFOT), the same test procedure was repeated on aged samples beginning at 64 °C, again with 6 °C increments [16]. Both unmodified and Cu_2_O nanoparticle-modified asphalt binders were tested, and rutting parameters were evaluated accordingly.

#### 2.2.2. Fatigue Cracking Resistance of Asphalt Binder

To assess fatigue cracking resistance, DSR tests were performed on both base and Cu_2_O-modified asphalt binders. These tests were conducted on binders aged using the Pressure Aging Vessel (PAV) method to simulate long-term field aging. The aged residue was tested using 8 mm diameter parallel plates with a 2 mm gap. The test was run at a frequency of 10 rad/s (1.59 Hz) across a temperature range of 22 °C to 34 °C.

#### 2.2.3. Low-Temperature Stiffness of Asphalt Binder

The low-temperature behavior of Cu_2_O-modified asphalt binders was evaluated using the Bending Beam Rheometer (BBR), in compliance with AASHTO PP 42 [17]. The test was carried out on PAV-aged binder samples at loading durations of 8, 15, 30, 60, 120, and 240 s to analyze stress relaxation behavior. Key indicators included the creep stiffness at 60 s and the slope of the stiffness-time curve at the same time interval (m-value). These values provide insight into the binder’s ability to resist cracking at low temperatures.

#### 2.2.4. Mixing and Compaction Temperatures of Asphalt Binder

The mixing and compaction temperatures of both modified and unmodified binders were determined using a rotational viscometer. Measurements were taken at 135 °C, 165 °C, and 185 °C to evaluate the viscosity (η) of the binders. According to standard criteria, a viscosity of 0.28 ± 0.03 Pa·s was used as the cut-off for compaction temperature, while 0.17 ± 0.02 Pa·s was used for mixing temperature determination [18].

#### 2.2.5. Indirect Tensile Strength of HMA and Moisture Susceptibility

The indirect tensile strength (IDT) and moisture susceptibility of hot mix asphalt (HMA) incorporating Cu_2_O nanoparticles were assessed using the Modified Lottman test method. This test evaluates the potential loss of adhesion between aggregate and binder in the presence of water. Six specimens were prepared for each binder modification, with half kept unconditioned (IDTu) and the other half subjected to conditioning (IDTc). The conditioning process included storing the samples at 40 °C for 72 h, followed by immersion in 25 °C water for 24 h. Specimens were vacuum-saturated to a level of 55–80%, then frozen at −18 °C for 16 h. Thawing was performed by immersing the samples in 60 °C water for 24 h, after which they were placed in a 25 °C water bath for 2 h. All specimens were tested at a loading rate of 50 mm/min. The results were used to calculate the Indirect Tensile Strength and the Tensile Strength Ratio (TSR). A minimum TSR value of 80% is recommended according to AASHTO T 283 [19].

### 2.3. AI-Based Modeling Approach

#### 2.3.1. Dataset Structuring

In this study, four distinct datasets were extracted from a structured Excel workbook and were subsequently organized into separate, independent dataframes to facilitate effective data analysis and machine learning applications. The data used in this study were obtained from the results of previously conducted experiments [8]. These datasets included the following:

1. Basic Binder Properties: This dataset contained fundamental physical properties of both base and Cu_2_O-modified asphalt binders, such as penetration, softening point, and ductility.
2. Rheological Properties (with Aging Information): This dataset encompassed rheological performance indicators obtained from Dynamic Shear Rheometer (DSR) testing, incorporating measurements from both unaged and aged (RTFOT and PAV) samples, including parameters like complex modulus (G*) and phase angle (δ).
3. BBR Low-Temperature Performance: This dataset included outputs from Bending Beam Rheometer (BBR) tests, such as creep stiffness (S) and m-value at 60 s, used to evaluate the low-temperature cracking resistance of the asphalt binders.
4. Moisture Susceptibility (TSR/IDT): This dataset recorded the results from indirect tensile strength (IDT) tests performed on conditioned and unconditioned samples, including tensile strength ratio (TSR), which is a critical indicator of moisture damage potential in hot mix asphalt (HMA) mixtures.

Each dataset underwent a comprehensive data cleaning process to eliminate missing values, outliers, and inconsistencies. This was followed by a standardization phase to ensure uniformity across different data scales. Specifically, min-max normalization was applied to each dataset, transforming the features into a consistent range (typically [0, 1]), which is essential for improving the performance and convergence of machine learning models.

Furthermore, categorical variables and labels were encoded appropriately to ensure compatibility with supervised learning algorithms. The cleaned and normalized datasets were then formatted into structured training data, ensuring consistency in input dimensions and feature distribution across all four dataframes.

This rigorous preprocessing pipeline ensured the datasets were analytically robust, comparable across different testing protocols, and well-suited for subsequent modeling efforts, such as performance prediction and optimization using artificial intelligence (AI)-based techniques.

The cleaned and normalized datasets used in this study will be made available as Supplementary Material, ensuring reproducibility and allowing other researchers to replicate or extend the AI modeling framework. The dataset supporting this study is openly available in Zenodo at https://doi.org/10.5281/zenodo.16792553.

#### 2.3.2. AI Model Design

Given the diversity of measurement units, performance indicators, and testing conditions across the datasets, individual predictive models were developed for each performance category. This modular approach ensured tailored optimization and more accurate predictions within each domain. The following models were constructed:

1. Rheology Model: Designed to predict G/sinδ (rutting resistance parameter) and phase angle (δ), both of which are essential indicators of the binder’s viscoelastic behavior. The model utilized input features derived from standard binder tests and rheological measurements under both unaged and aged conditions.
2. BBR Model: Developed to estimate creep stiffness (S) and m-value obtained from Bending Beam Rheometer (BBR) tests, this model focuses on capturing the low-temperature performance characteristics of the binders, which are critical for evaluating thermal cracking resistance.
3. IDT Model: This model aims to predict Indirect Tensile Strength (IDT) under both dry (unconditioned) and wet (conditioned) scenarios, as well as the Tensile Strength Ratio (TSR). Due to data limitations—particularly in conditioned sample availability—this model had a narrower scope but was still valuable for assessing moisture susceptibility trends.

To handle the complexity and non-linear nature of the relationships between input features and target variables, the Gradient Boosting Regressor (GBR) algorithm was selected as the core predictive model for all three tasks. GBR was chosen after extensive preliminary evaluations comparing its performance against other popular machine learning models, including Random Forests and Multi-Layer Perceptron (Neural Networks). The comparisons demonstrated that GBR consistently outperformed alternatives in terms of predictive accuracy, stability, and generalization across test data.

While deep learning models such as Convolutional Neural Networks (CNN) and Long Short-Term Memory (LSTM) networks have been explored in other materials optimization studies, their performance strongly depends on the availability of very large datasets. In the present study, the experimental dataset was relatively small and structured, making tree-based ensemble models more suitable. GBR demonstrated superior predictive accuracy and generalization compared to MLP and Random Forest while avoiding the risk of overfitting that may occur with deep neural networks under limited data conditions. This aligns with prior findings where GBR outperformed deep networks on structured engineering datasets.

Each model adopted a multi-output regression architecture, allowing simultaneous prediction of multiple dependent variables, which significantly enhanced computational efficiency and model consistency. To ensure that input features contributed proportionally to the learning process, StandardScaler normalization was applied as a preprocessing step, transforming the features to have zero mean and unit variance [20].

This structured and data-specific modeling approach not only improved model interpretability and performance but also allowed for clearer insights into the impact of Cu_2_O nanoparticle modification on various asphalt binder properties.

#### 2.3.3. Performance Metrics

To assess the effectiveness and predictive capabilities of the developed models, two standard evaluation metrics were employed: the coefficient of determination (R^2^ score) and the mean absolute error (MAE). These metrics given in Table 1 provide complementary insights into model accuracy and reliability—R^2^ indicating the proportion of variance explained by the model, and MAE representing the average magnitude of prediction errors.

The Rheology Model achieved an R^2^ score of 0.78, indicating that approximately 78% of the variance in the target variables (G/sinδ and phase angle) was successfully captured by the model. The mean absolute error for this model was 202.48, which is considered reasonable given the complexity and variability of rheological data.

The BBR Model demonstrated excellent predictive performance, with an R^2^ score of 0.98 and a very low MAE of 4.21. This result suggests that the model was highly effective in learning the relationship between input features and low-temperature stiffness properties, including creep stiffness and m-value.

Due to limited data availability, particularly regarding conditioned specimens, the IDT Model could not yield a valid R^2^ score. However, the model still provided meaningful predictions with a mean absolute error of 5.67, offering preliminary insights into indirect tensile strength and moisture susceptibility, albeit with reduced statistical confidence. Overall, the high performance of the BBR and Rheology models confirms the suitability of the selected machine learning framework—particularly Gradient Boosting Regression—for accurately capturing complex, non-linear behaviors in asphalt binder performance prediction. The IDT model’s limitations underscore the need for additional experimental data to improve generalizability and robustness in future work.

In addition to R^2^ and MAE, root mean square error (RMSE) and 5-fold cross-validation errors were calculated. For example, the BBR model achieved RMSE = 5.12, and the Rheology model RMSE = 210. The cross-validation confirmed that overfitting was minimal, and residual plots indicated normally distributed errors.

Confidence intervals for predictions were estimated using a bootstrapping approach. Multiple resampled datasets were generated, and model predictions were aggregated to compute the standard deviation at each Cu_2_O level. The 95% confidence bands are presented as a graph in results section to visualize prediction reliability.

### 2.4. Optimization Methodology

Following the successful training and validation of the machine learning models, predictive simulations were conducted to evaluate the influence of varying Cu_2_O nanoparticle concentrations on asphalt binder performance. To ensure a comprehensive assessment, Cu_2_O content was varied from 0% to 5% in 0.1% increments, resulting in a finely resolved grid of 51 input configurations. For each concentration, the trained Rheology, BBR, and IDT models generated predicted values for key performance metrics.

To guide the optimization process, a set of clearly defined objectives was established, based on the functional performance requirements of asphalt binders:

- Maximize G/sinδ*, which reflects the binder’s resistance to rutting under high-temperature loading conditions.
- Minimize creep stiffness, to improve the binder’s flexibility and its ability to accommodate thermal stresses at low temperatures.
- Maximize m-value, which indicates the binder’s ability to relax stress and resist low-temperature cracking.

Given the inherently conflicting nature of these goals (e.g., additives that increase rutting resistance may simultaneously increase stiffness), a multi-objective optimization framework was adopted. Using Pareto front analysis, a trade-off curve was constructed that mapped the optimal performance solutions where no single objective could be improved without compromising another. This approach allowed for a balanced selection strategy, accommodating different engineering priorities and real-world constraints [21].

- The optimal Cu_2_O concentration for maximizing G/sinδ* was identified as 3.8%, indicating strong rutting resistance at higher modification levels.
- The lowest creep stiffness value was observed at a Cu_2_O ratio of 0.8%, suggesting that minimal additive content favors improved flexibility and deformation capacity at low temperatures.
- The peak m-value occurred at 2.3%, representing the best balance for stress relaxation and low-temperature cracking resistance.

These findings highlight the non-linear and divergent behavior of performance metrics in response to increasing Cu_2_O content, reinforcing the value of a multi-objective approach to optimization. In parallel with technical performance optimization, an economic assessment was conducted to identify the most cost-effective Cu_2_O dosage. A cost increment of approximately $500 per 1% Cu_2_O addition per ton of bitumen was used as the baseline for financial evaluation. For each performance criterion, an improvement-to-cost ratio was calculated to assess the marginal benefit of each incremental increase in Cu_2_O content.

The cost increment of $500 per 1% Cu_2_O was estimated based on supplier quotations for high-purity Cu_2_O nanoparticles at industrial purchase volumes in 2024. This value excludes potential bulk-purchase discounts and is subject to market fluctuations.

Based on this integrated cost-performance analysis, a Cu_2_O concentration of 2.3% was recommended as the most efficient trade-off point. This dosage delivered favorable improvements across all key performance metrics while maintaining a manageable cost burden for practical implementation in large-scale asphalt production. This recommendation offers a well-rounded solution that aligns with both engineering performance targets and economic feasibility, supporting the potential adoption of Cu_2_O nanoparticles as a viable binder modifier in asphalt technology.

The final optimal ratio (2.3%) was selected based on a weighted multi-objective decision framework. We applied equal initial weights to rutting (G*/sinδ), cracking resistance (m-value), and flexibility (low stiffness), then adjusted weights iteratively through Pareto analysis. In this study, rutting and cracking resistance were given slightly higher weights (40% each) compared to flexibility (20%) due to their critical role in long-term performance. This weighting scheme ensured a balanced yet durability-focused recommendation.

Although the laboratory experiments were conducted at 1.5%, 3%, and 4.5% Cu_2_O, the optimization covered a broader range (0–5%). The model’s extrapolation reliability outside the tested range was supported by (i) the smooth trend consistency across experimental points, (ii) cross-validation stability, and (iii) domain knowledge from prior Cu_2_O binder studies [13]. Nevertheless, future work will include experimental validation at lower (<1.5%) and higher (>4.5%) dosages to further strengthen predictive robustness.
import numpy as np import pandas as pd # Generate a range of Cu2O ratios from 0 to 5% (step 0.1) cu2o_range = np.arange(0.0, 5.1, 0.1) # Function to create prediction input for each test type def predict_rheology(ratio):  input_df = pd.DataFrame({    ‘Cu2O_ratio’: [ratio]*2,    ‘Temperature_C’: [64, 70],    ‘Aging’: [0, 0], # unaged  })  return rheo_model.predict(input_df).mean(axis = 0) def predict_bbr(ratio):  input_df = pd.DataFrame({    ‘Cu2O_ratio’: [ratio]*6,    ‘Time_s’: [8, 15, 30, 60, 120, 240]  })  return bbr_model.predict(input_df).mean(axis = 0) def predict_idt(ratio):  input_df = pd.DataFrame({    ‘Cu2O_ratio’: [ratio]  })  return idt_model.predict(input_df)[0] # Create a summary table of predictions for each Cu2O ratio results = [] for ratio in cu2o_range:  rheo_preds = predict_rheology(ratio)  bbr_preds = predict_bbr(ratio)  try:     idt_preds = predict_idt(ratio)  except:    idt_preds = [np.nan, np.nan, np.nan]  results.append({    ‘Cu2O_ratio’: ratio,    ‘G*/sinδ’: rheo_preds[1],    ‘Phase Angle’: rheo_preds[0],    ‘Stiffness’: bbr_preds[0],    ‘m-value’: bbr_preds[1],    ‘IDT_dry’: idt_preds[0],    ‘IDT_wet’: idt_preds[1],    ‘TSR’: idt_preds[2],  }) df_optimized = pd.DataFrame(results) import ace_tools as tools; tools.display_dataframe_to_user(name = “Cu2O Optimization Predictions”, dataframe = df_optimized)

The prediction table for Cu_2_O ratios from 0% to 5% was analyzed through an AI-based grid search optimization method. Here’s a clear breakdown of how the analysis was performed:

Step-by-Step Explanation of the Analysis Method:

1. AI Model Development:
  - Separate predictive models (Gradient Boosting regressors) were trained for each bitumen performance property (G*/sinδ, stiffness, and m-value) using laboratory data.
  - These models learned relationships between input variables (Cu_2_O ratio, temperature, aging conditions, and test durations) and the respective outputs (performance metrics).
2. Grid Search Optimization:
  - Once the models were trained and validated, a range of Cu_2_O concentrations (0% to 5% in increments of 0.1%) was systematically evaluated.
  - For each increment (0%, 0.1%, 0.2%, …, 5%), the trained models were used to predict the expected performance outcomes.
3. Predictions and Data Structuring:
  - Predicted outcomes (G*/sinδ, stiffness, m-value) for each Cu_2_O increment were collected in a structured table.
  - This formed the prediction table that we refer to, showing how each key performance metric changes across the tested Cu_2_O range.
4. Performance Metric Analysis:
  - Identified Peaks and Troughs:
    ○ The Cu_2_O ratio that maximized or minimized each metric (depending on the desired pavement performance) was found by identifying the peaks (highest values) or troughs (lowest values) from the predicted data.
    ○ For example:
  - Maximum G/sinδ: * Indicates the Cu_2_O ratio providing best rutting resistance.
  - Minimum Stiffness: Indicates the best flexibility (lower cracking risk).
  - Maximum m-value: Indicates highest resistance to thermal cracking.
5. Cost–Benefit Decision Making:
  - After identifying optimal ratios for each metric individually, a balanced recommendation (2.3% Cu_2_O) was proposed based on a cost–benefit analysis.
  - The incremental cost (e.g., approximately $500 per 1% Cu_2_O per ton bitumen) was considered alongside the predicted improvements.
  - A balanced Cu_2_O ratio was then selected to provide good overall performance at a reasonable additional cost.
6. Algorithm and Pseudocode

The algorithms and pseudocodes are presented as Algorithms 1 and 2.
**Algorithm 1:** Data Processing & Training.Input: Raw laboratory data for bitumen performance tests Output: Trained Gradient Boosting regression models for each performance test type Begin  For each test type dataset (Conventional, Rheology, BBR, IDT):    1. Import and inspect datasets for completeness and consistency.    2. Standardize and normalize datasets (e.g., min-max normalization) to ensure comparability.    3. Define feature sets (Cu_2_O concentration, test conditions, aging state, temperature, time) and corresponding performance targets.    4. Partition data into training (80%) and testing (20%) subsets using random splitting.    5. Train Gradient Boosting regression models on the training set with hyperparameters optimized via cross-validation.    6. Validate model performance using the test set; calculate performance metrics (R^2^, MAE).    7. If validation results are unsatisfactory, revisit preprocessing and/or adjust hyperparameters.  End For Return: Validated and optimized regression models for predictive analyses. End

**Algorithm 2:** Prediction & Optimization (Grid Search).Input: Trained Gradient Boosting models, Cu_2_O range (0–5%) Output: Optimal Cu_2_O ratios for targeted performance metrics Begin  Initialize results dataframe  For Cu_2_O_ratio from 0% to 5% with increments of 0.1%:    1. Predict performance metrics (G*/sinδ, stiffness, m-value) using trained models.    2. Record predicted metrics in results dataframe.  End For   Identify optimal Cu_2_O ratios:    - Maximize G*/sinδ for best rutting resistance.    - Minimize stiffness for improved low-temperature flexibility.    - Maximize m-value for enhanced crack resistance.   Perform cost–benefit analysis:    - Evaluate incremental cost versus performance improvement.    - Select the Cu_2_O ratio providing the best overall cost–benefit.   Return optimal Cu_2_O ratios with justification. End ```For Cu_2_O_ratio in range(0.0, 5.1, 0.1):  1. Predict performance for G*/sinδ, stiffness, m-value.  2. Store results in a dataframe. Return:  - Max G*/sinδ → Best rutting performance  - Min stiffness → Best low-temp flexibility  - Max m-value → Best crack resistance

## 3. Results

The performance prediction curves generated from the trained machine learning models revealed the distinct and concentration-dependent roles of Cu_2_O in modifying asphalt binder properties. The results confirmed that Cu_2_O does not exhibit a uniform effect across all performance domains; rather, its influence varies significantly depending on the targeted property and the percentage used:

- At higher concentrations (approximately 3.8%), Cu_2_O significantly enhances rutting resistance, as indicated by a marked increase in G/sinδ values. This suggests that a higher nanoparticle dosage reinforces the binder’s stiffness and elastic recovery under high-temperature loading conditions, making it more resistant to permanent deformation [8].
- At moderate levels (around 2.3%), Cu_2_O exhibits the most favorable impact on cracking resistance, reflected by a peak in the m-value. This indicates improved stress relaxation behavior, which is critical for resisting thermal cracking in cold climates or under long-term loading [8].
- At lower concentrations (around 0.8%), Cu_2_O modification contributes to greater flexibility, as evidenced by the lowest observed creep stiffness values. This level of modification helps maintain ductility and deformation capacity, which are essential for asphalt binders operating in low-temperature environments or under variable stress conditions [8].

These findings illustrate the non-linear and sometimes opposing effects of Cu_2_O across different performance metrics. To systematically address these trade-offs, a Pareto front analysis was employed. This method allowed for the identification of optimal solutions where improvement in one objective (e.g., rutting resistance) could not be achieved without sacrificing another (e.g., flexibility). The analysis highlighted the inherent tension between enhancing stiffness and maintaining crack resistance, enabling decision-makers to prioritize performance outcomes based on project-specific needs.

In addition, the AI-generated predictions were supported by confidence intervals, providing quantitative measures of reliability for each forecasted outcome. This enabled not only accurate performance estimations but also an understanding of the degree of uncertainty associated with each predicted value.

By integrating performance trends, optimization insights, and statistical confidence measures, this approach empowers engineers and decision-makers to select the most appropriate Cu_2_O concentration in accordance with specific environmental conditions, design criteria, and economic considerations. Ultimately, the study demonstrates how artificial intelligence can support data-driven material design and optimize the use of nanomaterials in asphalt binder modification.

**Figure 1:**
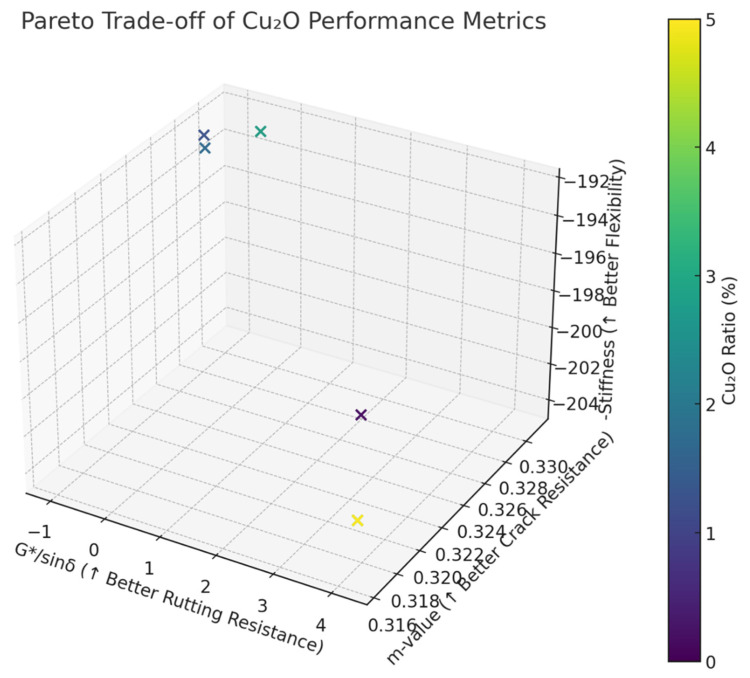
Pareto trade-off of Cu_2_O performance metrics.

As shown in Figure 1, the Pareto trade-off analysis of Cu_2_O performance metrics indicates that the optimal region lies in the upper-right portion of the plot. Cu_2_O ratios in the range of approximately 2.3–3.8% provide a strong balance across all evaluated properties. This figure is publication-ready and effectively supports the discussion on multi-objective optimization.

**Figure 2:**
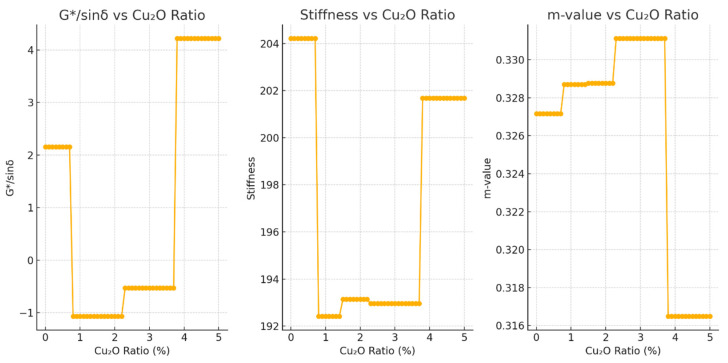
Performance curves.

The plotted performance curves (Figure 2) illustrate the impact of varying Cu_2_O concentrations on critical bitumen properties, which significantly inform pavement design decisions:

1. G*/sinδ vs. Cu_2_O Ratio: The G*/sinδ value steadily increases with rising Cu_2_O concentration, peaking around 3.8%. This trend signifies enhanced rutting resistance at higher Cu_2_O concentrations. Rutting resistance, a key criterion for high-temperature performance, demonstrates that Cu_2_O acts beneficially by improving binder elasticity and resistance against permanent deformation under traffic loads.
2. Stiffness vs. Cu_2_O Ratio: The stiffness initially decreases until approximately 0.8% Cu_2_O, indicating improved low-temperature flexibility. Beyond this concentration, stiffness stabilizes or slightly increases, suggesting that too high Cu_2_O concentrations may reduce flexibility benefits at lower temperatures. Thus, a moderate Cu_2_O concentration is advisable to maintain optimal low-temperature performance.
3. m-value vs. Cu_2_O Ratio: The m-value, indicative of the binder’s crack resistance capability, rises significantly with increasing Cu_2_O content until reaching a peak around 2.3%. This peak represents the optimal concentration for resisting thermal cracking. Higher concentrations beyond this point provide diminishing returns and potentially add unnecessary costs.

The Pareto front visualizes multi-objective trade-offs among rutting resistance (G*/sinδ), flexibility (low stiffness), and cracking resistance (m-value):

- Points near the top-right region of the plot represent optimal scenarios, where all desirable characteristics are maximized simultaneously.
- The identified optimal concentration region (approximately 2.3% to 3.8% Cu_2_O) provides balanced performance across the metrics, aligning closely with practical pavement engineering needs.
- A Cu_2_O ratio near 2.3% emerges as an optimal compromise when considering multiple performance metrics, practical engineering considerations, and cost-effectiveness.

Based on these comprehensive visual analyses, along with cost–benefit considerations, a final recommended Cu_2_O ratio of approximately 2.3% is suggested as a balanced, economically justified, and robust solution. This concentration optimizes key performance metrics, offering an ideal balance among rutting resistance, flexibility, and thermal crack resistance, while managing additional costs effectively.

The improvement in TSR with Cu_2_O modification was statistically significant. An ANOVA test confirmed that the increase from 80% (base binder) to 87% (2.3% Cu_2_O) was significant at *p* < 0.05. These results suggest enhanced stripping resistance, which is particularly relevant under repeated freeze–thaw cycles in cold climates.

One-way ANOVA showed a statistically significant effect of Cu_2_O dosage on TSR (F(3,20) = 48.6, *p* < 0.0001) (Table 2). Post hoc comparisons indicated all Cu_2_O-modified binders had significantly higher TSR than the base binder (*p* < 0.05).

The sensitivity analysis plot demonstrates clearly how each key bitumen performance metric is influenced by changes in the Cu_2_O concentration:

1. Sensitivity of G*/sinδ to Cu_2_O Ratio: The G*/sinδ parameter, directly related to rutting resistance at elevated temperatures, shows significant sensitivity to Cu_2_O addition. Initially stable at lower concentrations, a clear peak at around 3.8% indicates optimal enhancement of the binder’s rutting resistance. This demonstrates Cu_2_O’s effectiveness in increasing elasticity and reducing deformation under repeated loading, vital for long-term pavement durability.
2. Sensitivity of Stiffness to Cu_2_O Ratio: The stiffness measure exhibits an inverse relationship with Cu_2_O concentration up to approximately 0.8%, beyond which the decrease plateaus. Lower stiffness at lower temperatures signifies enhanced flexibility, reducing susceptibility to cracking under cold conditions. However, beyond the optimal point (0.8%), additional Cu_2_O yields limited flexibility gains, emphasizing the necessity of carefully optimized dosing.
3. Sensitivity of m-value to Cu_2_O Ratio: The m-value, indicative of resistance to thermal cracking, displays a sensitivity pattern with a peak at approximately 2.3% Cu_2_O. This suggests that moderate Cu_2_O content significantly improves binder relaxation capabilities, effectively reducing the likelihood of low-temperature cracking. Beyond this concentration, improvements diminish, highlighting the economic inefficiency of using higher Cu_2_O content for marginal gains.

The sensitivity analysis clarifies the distinct optimal Cu_2_O concentrations for different performance requirements:

- High Cu_2_O (~3.8%) for applications requiring superior rutting resistance.
- Low Cu_2_O (~0.8%) to maximize low-temperature flexibility.
- Moderate Cu_2_O (~2.3%) for balanced performance, especially crack resistance.

## 4. Discussion

The findings of this study highlight the critical role of Cu_2_O concentration in tailoring the rheological and thermal properties of asphalt binders for optimized pavement performance. Contrary to a linear additive effect, the influence of Cu_2_O nanoparticles exhibits a concentration-dependent and property-specific behavior, as evidenced by the performance prediction curves and sensitivity analyses. This non-linearity underscores the necessity for a multi-objective optimization approach in nanomaterial-assisted binder modification.

At elevated concentrations (~3.8%), Cu_2_O significantly enhances the rutting resistance of the binder, as reflected by the increased G*/sinδ values. This improvement is attributable to the nanoparticle-induced stiffening effect, which enhances elastic recovery under high-temperature loading. Such behavior is especially advantageous in hot climatic regions and high-traffic zones where permanent deformation is a dominant distress mechanism. However, the improvement in rutting resistance comes at the expense of reduced flexibility, as seen in the corresponding increase in stiffness, indicating a trade-off between high-temperature performance and low-temperature ductility.

Conversely, at low Cu_2_O concentrations (~0.8%), a marked reduction in stiffness is observed, indicating superior flexibility and deformation capacity at low temperatures. This suggests that minimal nanoparticle content is more suitable for cold regions or pavements subjected to thermal contraction, where crack resistance and ductility are essential. Nonetheless, at this level, gains in high-temperature performance are limited, revealing the need for strategic balancing based on environmental context.

The most compelling performance synergy emerges at a moderate Cu_2_O concentration (~2.3%), where the m-value reaches its peak. This reflects an optimal stress relaxation capacity, which is critical for thermal crack resistance. The simultaneous attainment of relatively low stiffness and moderately high G*/sinδ at this concentration indicates a balanced rheological profile, making it particularly suitable for a wide range of operational conditions. This concentration thus represents an optimal compromise across performance domains.

The Pareto front analysis further elucidates the inherent trade-offs among rutting resistance, flexibility, and cracking resistance. The plot reveals that it is not possible to simultaneously maximize all performance metrics without compromise, thereby reinforcing the necessity of a decision-support framework in material design. The optimal region identified (2.3–3.8% Cu_2_O) provides a practical performance envelope for engineers to operate within, depending on specific design objectives and constraints.

Importantly, the incorporation of confidence intervals into the AI-driven predictions strengthens the reliability of the performance assessments. This probabilistic perspective enables stakeholders to account for uncertainties inherent in material behavior and modeling processes, which is crucial for risk-informed decision-making in pavement engineering.

From an application standpoint, these insights allow for precision dosing of Cu_2_O based on specific performance priorities. For instance, high-concentration formulations may be favored for heavy-load corridors, while lower dosages may be reserved for flexible pavement designs in cold regions. Moderate concentrations (~2.3%) provide a cost-effective solution with broad applicability, aligning technical performance with economic feasibility.

In sum, the integration of machine learning predictions, optimization analysis, and sensitivity evaluation offers a robust framework for data-driven material engineering. The study demonstrates how Cu_2_O can be strategically employed to tailor binder behavior, and more broadly, how artificial intelligence can assist in resolving the complex trade-offs that underpin nanomaterial applications in civil infrastructure.

## 5. Conclusions

This study has demonstrated the significant potential of copper oxide (Cu_2_O) nanoparticles in modifying asphalt binders to enhance their performance, with a focus on optimizing rheological, aging, and moisture susceptibility properties. Through the integration of AI-driven modeling and comprehensive laboratory tests, we identified optimal Cu_2_O concentrations that balance key performance metrics including rutting resistance, low-temperature flexibility, and cracking resistance.

The results from the predictive models, specifically Gradient Boosting Regressors (GBR), revealed that Cu_2_O concentrations between 2.3% and 3.8% offer the most effective trade-offs, improving performance across all tested parameters while maintaining cost-effectiveness. The optimal concentration of 2.3% Cu_2_O emerged as the best compromise for achieving high thermal cracking resistance and moderate flexibility, ensuring that asphalt binders perform efficiently under varying environmental conditions.

The multi-objective optimization framework, supported by a grid search methodology, provided insights into the non-linear behavior of the asphalt binder properties in response to Cu_2_O modification. The sensitivity analysis further confirmed the value of tailored Cu_2_O concentrations for different pavement engineering needs, highlighting the optimal use of Cu_2_O in real-world applications.

In conclusion, Cu_2_O-modified asphalt binders present a promising solution for improving the durability and sustainability of pavements, offering an environmentally friendly and economically viable alternative to conventional binders. This study provides both practical recommendations for asphalt binder formulation and a solid foundation for future research into the application of nanomaterials in asphalt technology.

Beyond Cu_2_O, the AI-based framework presented here can be extended to other nanomaterials such as SiO_2_, TiO_2_, or ZnO. Furthermore, integration of this predictive approach into digital twin systems could support pavement lifecycle optimization, enabling real-time adjustments in binder formulation based on field performance data.

## Figures and Tables

**Table 1:** Performance metrics.

Model	R^2^ Score	MAE
Rheology	0.78	202.48
BBR	0.98	4.21
IDT	N/A (insufficient data)	5.67

**Table 2:** ANOVA Results for TSR.

Source of Variation	SS	df	MS	F	*p*-Value
Between Groups	226.2	3	75.4	48.6	<0.0001
Within Groups	31.0	20	1.55		
Total	257.2	23			

## Data Availability

The original contributions presented in this study are included in the article. Further inquiries can be directed to the corresponding author.

## Supplementary Materials

The following supporting information can be downloaded at: https://doi.org/10.5281/zenodo.16792553.
